# Discovery of a Eukaryotic Pyrroloquinoline Quinone-Dependent Oxidoreductase Belonging to a New Auxiliary Activity Family in the Database of Carbohydrate-Active Enzymes

**DOI:** 10.1371/journal.pone.0104851

**Published:** 2014-08-14

**Authors:** Hirotoshi Matsumura, Kiwamu Umezawa, Kouta Takeda, Naohisa Sugimoto, Takuya Ishida, Masahiro Samejima, Hiroyuki Ohno, Makoto Yoshida, Kiyohiko Igarashi, Nobuhumi Nakamura

**Affiliations:** 1 Department of Biomaterials Sciences, Graduate School of Agriculture and Life Sciences, The University of Tokyo, Tokyo, Japan; 2 Department of Environmental and Natural Resource Science, Tokyo University of Agriculture and Technology, Tokyo, Japan; 3 Department of Biotechnology and Life Science, Tokyo University of Agriculture and Technology, Tokyo, Japan; 4 Advanced Low Carbon Technology Research and Development Program (ALCA), Japan Science and Technology Agency (JST), Tokyo, Japan; Instituto de Tecnologica Química e Biológica, UNL, Portugal

## Abstract

Pyrroloquinoline quinone (PQQ) is a redox cofactor utilized by a number of prokaryotic dehydrogenases. Not all prokaryotic organisms are capable of synthesizing PQQ, even though it plays important roles in the growth and development of many organisms, including humans. The existence of PQQ-dependent enzymes in eukaryotes has been suggested based on homology studies or the presence of PQQ-binding motifs, but there has been no evidence that such enzymes utilize PQQ as a redox cofactor. However, during our studies of hemoproteins, we fortuitously discovered a novel PQQ-dependent sugar oxidoreductase in a mushroom, the basidiomycete *Coprinopsis cinerea*. The enzyme protein has a signal peptide for extracellular secretion and a domain for adsorption on cellulose, in addition to the PQQ-dependent sugar dehydrogenase and cytochrome domains. Although this enzyme shows low amino acid sequence homology with known PQQ-dependent enzymes, it strongly binds PQQ and shows PQQ-dependent activity. BLAST search uncovered the existence of many genes encoding homologous proteins in bacteria, archaea, amoebozoa, and fungi, and phylogenetic analysis suggested that these quinoproteins may be members of a new family that is widely distributed not only in prokaryotes, but also in eukaryotes.

## Introduction

**Figure 1:**
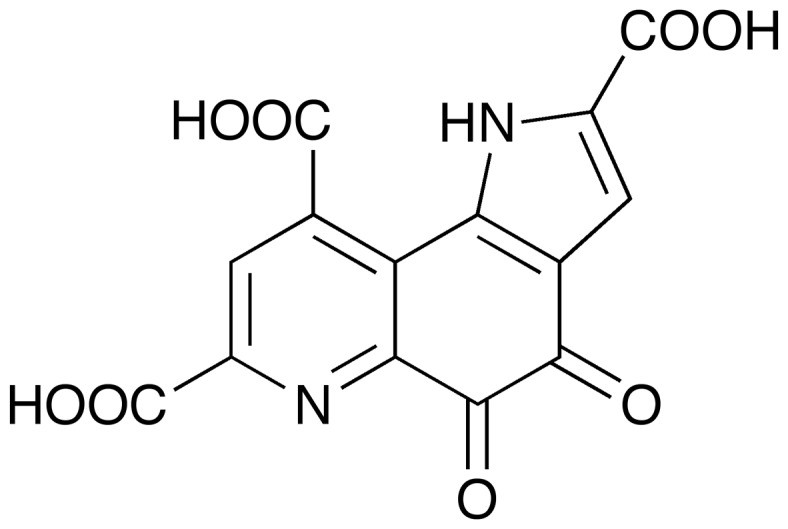
Chemical structure of PQQ.

Pyrroloquinoline quinone (Figure 1, PQQ), a cofactor of prokaryotic dehydrogenases, was discovered as a redox cofactor of bacterial glucose dehydrogenase in 1964 [1] Its chemical structure was reported after its isolation from primary alcohol dehydrogenase of methylotrophic bacteria [2]. Some prokaryotic organisms are able to synthesize PQQ, whereas others require an exogenous source. The enzyme group to which the PQQ cofactor belongs is the quinoprotein group [3]–[5], and all PQQ-dependent quinoproteins reported to date have a characteristic propeller-fold superbarrel structure [3], [5]. Bacterial PQQ-dependent dehydrogenases which have an eight-bladed structure contain the characteristic amino acid sequence, which has been usually used to identify PQQ-dependent proteins. Although PQQ is physiologically important in plants [6] as well as mammals (including humans) [7]–[10], there has been no evidence showing PQQ as a cofactor of any eukaryotic enzyme so far.

A new class, named “Auxiliary Activities” (AA), was recently introduced in the database of carbohydrate-active enzymes (CAZy) to cover the full extent of the lignocellulose breakdown machinery [11]. This class covers a broad group of catalytic modules associated with plant cell wall degradation. The members of the AA family 8 consist of a cytochrome domain of spectral class *b* (protoheme IX); the first example of this family was found as a domain of cellobiose dehydrogenase (CDH; EC 1.1.99.18) isolated from the wood-rotting basidiomycete *Phanerochaete chrysosporium*, in which the cytochrome domain was linked to a flavin-containing AA family 3 flavin-containing sugar dehydrogenase domain [12]. We are interested in the AA family 8 hemoproteins because the heme domain is the first cytochrome *b*-type heme bound through Met/His with a primarily β-structure [13], and appears to be unrelated to any known protein other than the cytochrome domain of CDHs.

**Figure 2:**
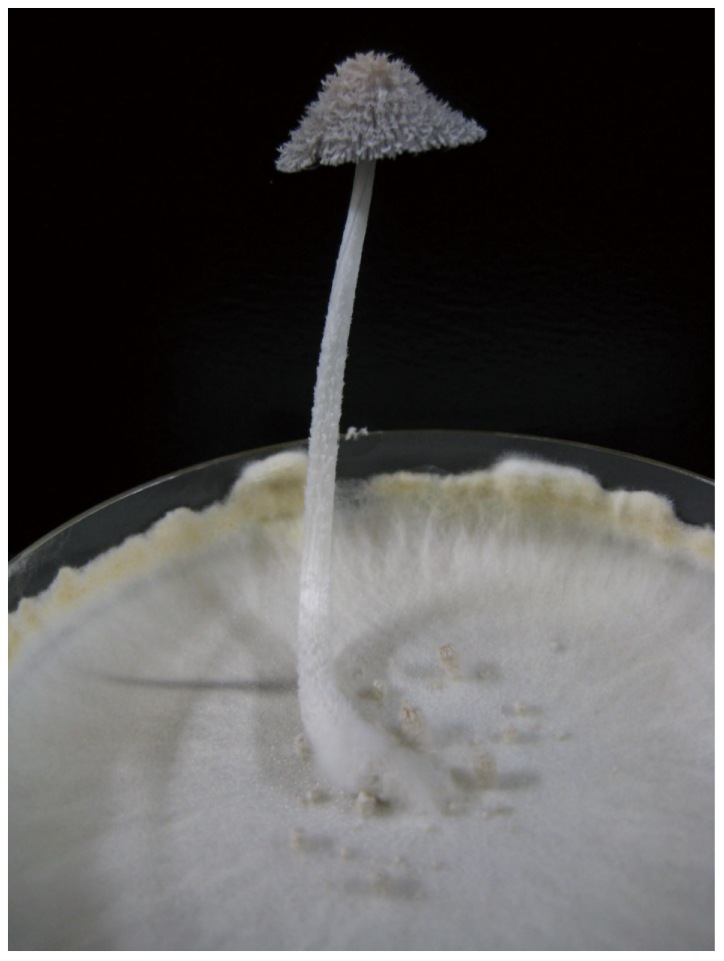
Fruiting body of *Coprinopsis cinerea*.

The basidiomycete *Coprinopsis cinerea* (formerly known as *Coprinus cinereus*, Figure 2) produces many extracellular oxidoreductases, including peroxidases and laccases, as well as cellulolytic enzymes, and has many putative oxidoreductase genes. To identify novel proteins with this unique heme *b*-containing cytochrome domain, the amino acid sequence of the cytochrome domain of CDH from *P. chrysosporium* was subjected to BLAST search using the *C. cinerea* Okayama 7 (#130) genome database at the Broad Institute. We fortuitously discovered a novel PQQ-dependent protein that belongs to a category that is different from the known PQQ-quinoprotein family during a search for AA family 8 cytochrome domains.

## Materials and Methods

### Materials

*Coprinopsis cinerea* strain 5338 was kindly provided by Dr. Yasuhiro Ito (National Food Research Institute, Ibaraki, Japan) [14]. Pyrroloquinoline quinone was purchased from Sigma-Aldrich (Tokyo, Japan). Bovine heart cytochrome *c* was purchased from Wako Pure Chemical Industries (Osaka, Japan).

### Cloning and transcript analysis

*C. cinerea* was grown in Kremer and Wood medium [15] containing 2% Avicel (Merck, Whitehouse Station, NJ) for 12 d, and then total RNA was extracted using the RNeasy Plant Mini Kit (Qiagen, Hilden, Germany). First-strand cDNA was synthesized using SuperScript III reverse transcriptase (Invitrogen, Carlsbad, CA) with an oligo(dT) primer (Takara Bio, Shiga, Japan). PCR using KOD plus version 2 DNA polymerase (Toyobo, Osaka, Japan) with the synthesized first-strand cDNA as a template was primed with oligonucleotides flanking the predicted translational start and stop codons (20 nucleotides upstream, 5′-TCGGGACCGACCACGAACG-3′; 63 nucleotides downstream, 5′-CGATTCTGTCTTGAAGCCCGACT-3′). The PCR products were subcloned into the pGEM-T Easy vector (Promega, Madison, WI) followed by sequencing with a 3130 Genetic Analyzer (Applied Biosystems, Foster City, CA). The nucleotide sequence reported in this paper has been deposited in the DDBJ database under accession number AB901366.

### Sequence analysis

The *C. cinerea* genome database (http://www.broadinstitute.org/annotation/genome/coprinus_cinereus/MultiHome.html) was searched with the amino acid sequence of CDH from *P. chrysosporium* (accession no. AAB92262) using the TBLASTN algorithm [16], [17]. The search was carried out with standard settings and the BLOSUM 62 matrix. The amino acid sequences of candidate genes were scanned for the presence of signal peptides using SignalP version 4.1 software [18] at the Center for Biological Sequence Analysis (http://www.cbs.dtu.dk/services/SignalP/) and searched against the protein database at the National Center for Biotechnology Information (http://blast.ncbi.nlm.nih.gov/Blast.cgi). The homology structure model was constructed using Protein Homology/analogy Recognition Engine (Phyre) version 2.0 (http://www.sbg.bio.ic.ac.uk/phyre2/) [19]. The *Cc*SDH model was used for preparing a figure in which PQQ of sGDH from *Acinetobacter calcoaceticus* and heme in the cytochrome domain of *P. chrysosporium* CDH (PDBID 1CRU and 1D7C, respectively) were superimposed to the model. The structure based alignment was created during the process of homology modeling using the Phyre2 server, and the corresponding figure was prepared using ESPript ver. 2.3 [20].

### Production and purification of recombinant protein in *P. pastoris*

The expression vector was constructed as described [21], [22]. Two different oligonucleotide primers were designed based on the nucleotide sequence of the mature protein to allow for ligation into the *Eco*RI and *Not*I sites of the *Pichia* expression vector pPICZα (Invitrogen, Carlsbad, CA): 5′ primer (5′-TTGAATTCCAAGGCTCTCCCACTCAGTG-3′) and 3′ primer (5′-TTGCGGCCGCTATGCAGGAACACACTGAGAGTACC-3′). PCR was carried out using the primer pair with KOD plus version 2 DNA polymerase (Toyobo, Osaka, Japan) and the first-strand cDNA synthesized above as a template. The PCR product was subcloned into the pGEM-T Easy Vector (Promega, Madison, WI) followed by sequencing with a 3130 Genetic Analyzer (Applied Biosystems, Foster City, CA). The target gene was digested with *Eco*RI and *Not*I and ligated into the corresponding restriction sites of the pPICZαa vector. The nucleotide sequence of the inserted cDNA was confirmed by sequence analysis. Approximately 10 µg of the DNA construct in pPICZα was linearized with Bpu 1102I (Takara, Japan) prior to transformation into *P. pastoris.* Electroporation, selection of the transformant, and production of recombinant protein were carried out using an EasySelect *Pichia* expression kit (version G; Invitrogen, Carlsbad, CA) according to the manufacturer’s instruction. The PQQ domain of the recombinant protein was constructed by deleting the coding regions for the heme domain and the carbohydrate-binding module (CBM) domain of the recombinant protein by PCR using pPICZα containing the full-length gene as a template. The two oligonucleotide primers used to truncate the heme domain (221 amino acids from the N-terminus) were 5′-CGAGAAAAGAACCTTCGTCTCTTGC-3′ as the 5′ primer and 5′-GCAAGAGACGAAGGTTCTTTTCTCG-3′ as the 3′ primer; these primers included the α-factor signal sequence of the pPICZα vector upstream of the recombinant protein gene and the gene coding for the amino acids after residue T222. For truncation of the CBM domain, a stop codon was introduced by using the following primers: 5′- CATCATCAAGCGCTAGTCCGGCCCTATTGTTCAGC-3′ (5′ primer) and 5′- GCTGAACAATAGGGCCGGACTAGCGCTTGATGATG-3′ (3′ primer). The underlined codons represent mismatches that will introduce a stop codon and prevent the translation of the last 77 C-terminal amino acids, including the CBM domain. The mutations were confirmed by DNA sequencing, and the recombinant plasmid was transformed into *P. pastoris* for subsequent protein expression according to the same protocol used for the full-length recombinant protein.

The culture was centrifuged (30 min, 1,500×g), and the crude proteins (including the recombinant protein) were precipitated with ammonium sulfate (70% saturation) from the cell-free culture. The precipitates were dissolved in 20 mM Tris-HCl buffer containing 1 M ammonium sulfate (pH 8.0). The crude proteins were fractioned on a Toyopearl Phenyl-650S column equilibrated with 20 mM Tris-HCl buffer containing 1 M ammonium sulfate (pH 8.0). The recombinant protein was eluted using a linear reverse gradient to 20 mM Tris-HCl buffer (pH 8.0). The fraction containing the recombinant protein was collected and equilibrated against 20 mM Tris-HCl buffer (pH 8.5). The solution was loaded on a Toyopearl DEAE-650S column equilibrated with 20 mM Tris-HCl (pH 8.5). The enzyme was eluted from the column using a linear gradient in the same buffer (0 to 500 mM NaCl). The purity was confirmed by SDS-PAGE analysis on 12% polyacrylamide gels, and the N-terminal amino acid sequence was determined with a protein sequencer (model 491 cLC; Applied Biosystems, Foster City, CA) as described. The recombinant protein concentration in the solution was estimated from the absorbance at 420 nm (ε_420_ = 130 mM^−1^cm^−1^). Deglycosylation of the recombinant protein was performed using endo-β-*N*-acetylglucosaminidase H (endo-H) as described [21], [22].

### Enzyme activity

The enzyme activity was assayed using cytochrome *c* as an electron acceptor [23], [24]. The assay was performed by photometric monitoring of the time-dependent reduction of 50 µM cytochrome *c* at 550 nm (ε_550_ = 17.5 mM^−1^ cm^−1^) at 30°C. One activity unit (U) corresponds to the amount of enzyme that can convert 1 µmol of substrate per minute. The substrate specificity of the recombinant protein (40 nM) was assessed using 1 mM D-, L-allose, D-, L-galactose, D-, L-glucose, D-, L-gulose, D-, L-mannose, D-, L-talose, D-, L-arabinose, D-, L-lyxose, D-, L-xylose, D-, L-fructose, D-, L-tagatose, L-sorbose, cellobiose, maltose, sophorose, trehalose, D-, L-fucose, L-rhamnnose, N-acetyl-D-glucosamine, D-glucosamine, and D-glucosone. To obtain the kinetic parameters of oxidation for L-gulose, D-arabinose, D-lyxose, L-fucose, and D-glucosone, the assay was performed with various concentrations of the saccharides toward 50 nM recombinant protein in 50 mM Tris-HCl buffer (pH 8.5) containing 1 µM PQQ and 1 mM CaCl_2_. To determine the Michaelis constant (*K*_m_) and catalytic rate (*k*_cat_), the experimental data were fitted to the Michaelis-Menten equation.

### Isothermal titration calorimetry (ITC)

ITC experiments were carried out at 25°C with a MicroCal VP-ITC (GE Healthcare, Northampton, MA, USA). A sample of the purified PQQ domain of the recombinant protein was prepared by dialysis against ITC buffer (20 mM sodium acetate, pH 6.0, 1 mM CaCl_2_). The concentration of the PQQ domain of the recombinant protein was determined from the UV absorbance at 280 nm using the calculated molar extinction coefficient 7.241×10^4 ^M^−1^cm^−1^. PQQ was dissolved in the same ITC buffer, and the concentration was measured based upon the absorbance at 257 nm using the molar extinction coefficient [25] 2.15×10^4 ^M^−1^cm^−1^. Solutions were de-gassed for 1 min using a vacuum degasser immediately prior to experimentation. The titration sequence consisted of an initial injection of 2 µL of PQQ (46.3 µM) followed by 69 additional injections of 4 µL of PQQ at 220 s intervals into a calorimeter cell containing the PQQ domain of the recombinant protein solution (6.73 µM) stirred at 300 rpm. Baseline-subtracted data were fitted according to Marquardt’s single-site model using Origin ITC Analysis software (Microcal Software, Northampton, MA) to obtain stoichiometric and thermodynamic parameters.

### Construction of a phylogenetic tree

The National Center for Biotechnology Information protein database was searched for amino acid sequences corresponding to the catalytic domain of the recombinant protein using the PSI-BLAST algorithm. All searches were performed with standard settings and the BLOSUM 62 matrix. The sequences obtained and the sequences of known quinoproteins (PQQ-dependent methanol dehydrogenases, ethanol dehydrogenases, soluble glucose dehydrogenases, quinohemoprotein alcohol dehydrogenases, polyvinyl alcohol dehydrogenase, membrane-bound glucose dehydrogenase, membrane-bound alcohol dehydrogenase, and aldose sugar dehydrogenases) were subjected to alignment analysis using MAFFT (ver. 6.85) [26], [27]. Non-conserved regions were trimmed using SeaView (ver. 4.4.2) software. The phylogenetic tree was generated from the trimmed sequences using ClustalX (ver. 2.1) with the neighbor-joining method (Bootstrap value, 1000).

## Results

**Figure 3:**
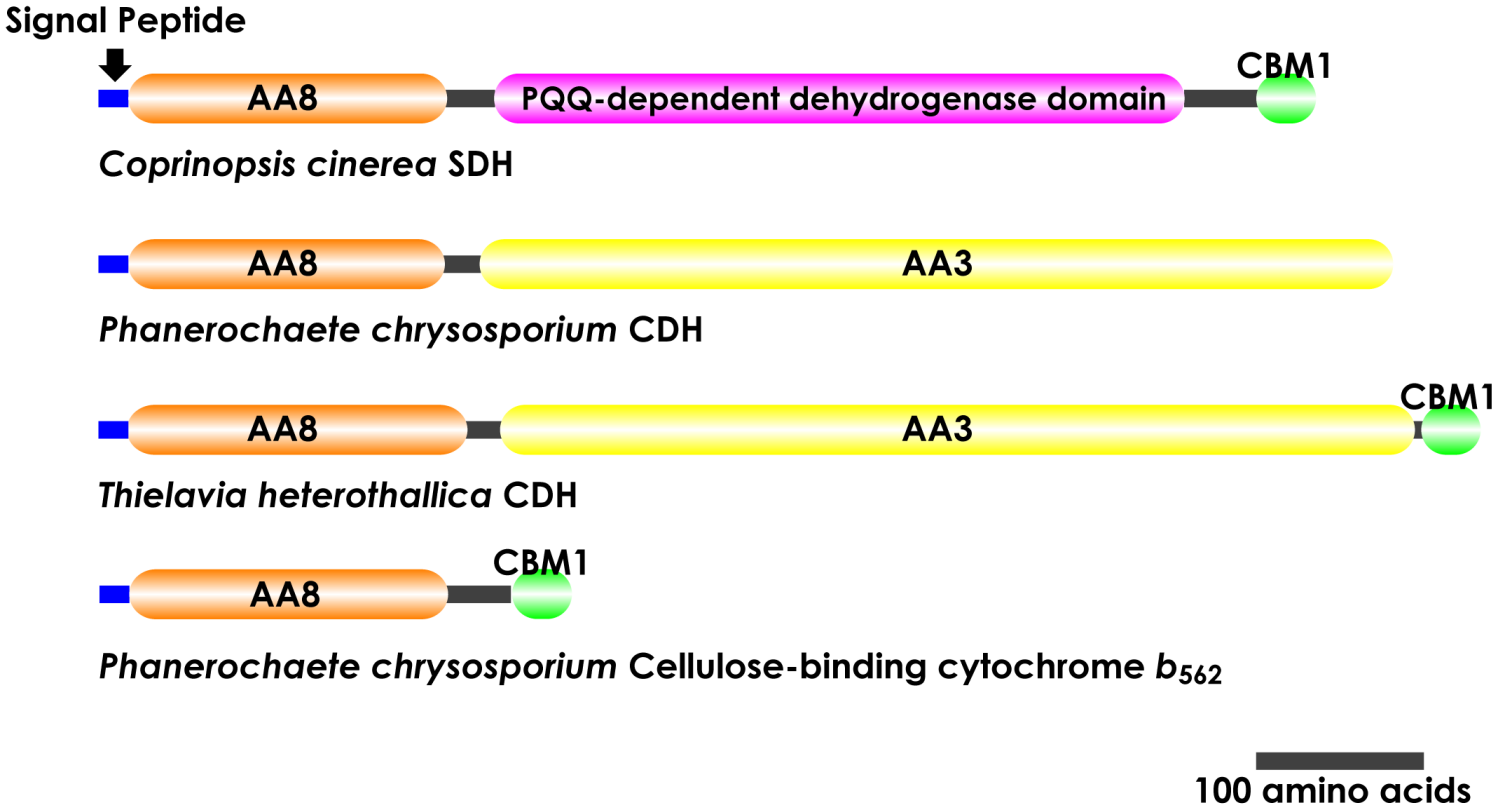
Domain organization of *Cc*SDH, CDH from *P. chrysosporium*, CBM1-carrying CDH from *T. heterothallica*, and cellulose-binding cytochrome *b*_562_ from *P. chrysosporium*. Abbreviations: AA3, Auxiliary Activities (AA) family 3 enzymes defined as flavoproteins containing a flavin-adenine dinucleotide (FAD)-binding domain; AA8, AA family 8 enzymes with the cytochrome domain of spectral class *b*; CBM1, family 1 carbohydrate-binding module.

As a result of the BLAST search, several genes, including genes homologous to the cytochrome domain of CDH, were identified in the *C. cinerea* genome, and we cloned one of them (Chromosome 6∶1274274–1277156). RT-PCR using total RNA of *C. cinerea* was performed to obtain the cDNA of the whole protein. The cDNA consisted of 2883 bp, which included an open reading frame for 726 amino acids. Based on the analysis of the predicted amino acid sequence using the SignalP 4.1 server [18], the N-terminal 18 amino acid sequence was identified as a signal peptide, suggesting that the protein is secreted extracellularly. Homology searches of the amino acid sequence in the NCBI protein database revealed that the protein was composed of an N-terminal AA family 8 cytochrome domain and a C-terminal cellulose-binding domain belonging to the CBM family 1 as shown in Figure 3. In addition to these domains, the protein contains an unknown domain, which has a low similarity to glucose/sorbosone dehydrogenases, different than the AA family 3 flavin domain seen in CDHs.

**Figure 4:**
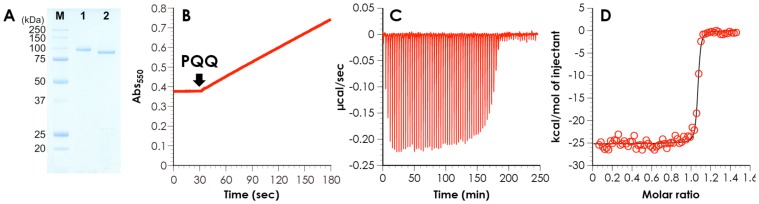
A, SDS-PAGE of purified (lane 1) and deglycosylated (lane 2) *Cc*SDH. B, Effect of PQQ on cytochrome *c*-reducing activity of *Cc*SDH with L-fucose as a substrate. For the activity measurement in B, 40 nM *Cc*SDH was incubated with 20 mM L-fucose, 50 µM cytochrome *c* and 1 µM PQQ in HEPES buffer (pH 7.0), and the reduction of cytochrome *c* was monitored based upon the increase of absorption at 550 nm. C and D, Isothermal titration calorimetry analysis of the PQQ domain of *Cc*SDH and PQQ with the raw data (C) and plots of the integrated peaks (D). The PQQ domain of *Cc*SDH (6.73 µM) was titrated with an initial injection of 2 µL of PQQ (46.3 µM) followed by 69 consecutive injections of 4 µL of PQQ in the presence of 1 mM CaCl_2_ and 20 mM sodium acetate (pH 6.0). The black line indicates the best fit to a single-site model.

The recombinant protein was named *C. cinerea* sugar dehydrogenase (*Cc*SDH) because of its oxidative activity towards various sugars. This enzyme was heterologously expressed in the methylotrophic yeast *Pichia pastoris* and purified from the culture solution using a two-step column chromatography. The purified enzyme yielded a single band at a molecular mass of 98±8 kDa on SDS-PAGE (Figure 4A). The molecular mass measured was larger than that estimated from the amino acid sequence (77 kDa), suggesting that *Cc*SDH is *N*- and *O*-glycosylated, as predicted by the NetNGlyc and NetOGlyc servers. This conclusion was confirmed by a decrease of the apparent molecular weight following treatment with endo-glycosidase H as shown in Figure 4A. In anticipation that the unknown domain would have redox activity based on the electron transfer ability of the cytochrome domain, we tested NAD(P), FAD, and PQQ as cofactors for two-electron oxidation reaction of various sugars, alcohols, aldehydes, and their derivatives. The assay was carried out by monitoring the rate of cytochrome *c* reduction (similar to the CDH assay [23], [24]). Interestingly, when various sugars (including D-glucosone, L-fucose, and some rare sugars) were used as substrates, the reduction of cytochrome *c* was observed only in the presence of PQQ. *Cc*SDH had catalytic activity toward L-galactose, L-gulose, D-talose, D-arabinose, D-lyxose, L-fucose, and D-glucosone in the presence of PQQ and CaCl_2_. *Cc*SDH shows little or no activity toward abundant sugars such as D-glucose, D-fructose, and cellobiose. The steady-state kinetic parameters for L-gulose, D-arabinose, D-lyxose, L-fucose, and D-glucosone were measured using the cytochrome *c* assay under optimal condition as summarized in Table 1. Because D-glucosone has the reductive activity of cytochrome *c*, we determined the initial velocity in an enzymatic reaction after subtracting the rate of cytochrome *c* reduction by D-glucosone itself from an apparent rate of cytochrome *c* reduction. The catalytic efficiency measured was in the following order: D-glucosone>L-fucose>D-arabinose>L-gulose>D-lyxose. D-glucosone exhibited the highest catalytic efficiency, *k*_cat_/*K*_m_, calculated to be 9.37×10^3^ s^−1^M^−1^. The *K*_m_ values for these sugars appear to be large. However, considering the fact that the PQQ-dependent sugar dehydrogenases, such as soluble glucose dehydrogenase from *Acinetobacter calcoaceticus* (sGDH) have a large *K*_m_ value (25 mM) for glucose, a physiological substrate [28], the *K*_m_ value for D-glucosone (7.9 mM) with *Cc*SDH is reasonable for a quinoprotein. Figure 4B shows the time-course of the absorbance at 550 nm based upon the reduced cytochrome *c* for L-fucose as a typical example. When *Cc*SDH was added to the assay solution without PQQ, no catalytic activity was observed. Upon addition of PQQ, the absorbance immediately increased. To determine the binding constant of the protein to PQQ, the PQQ domain alone was expressed in the *Pichia* expression system and the binding constant was estimated by ITC as shown in Figure 4C. The dissociation constant (*K*_d_) was 1.11 nM, and the molar ratio of PQQ to the domain was 1∶1 (Figure 4D).

**Table 1:** Kinetic parameters of CcSDH for various monosaccharides.

	*k*_cat_ (s^−1^)	*K*_m_ (mM)	*k*_cat_/*K*_m_ (×10^3^ s^−1^M^−1^)
D-glucosone	74.1 (±1.4)	7.9 (±0.3)	9.37
L-fucose	56.4 (±1.8)	24.8 (±1.2)	2.27
D-arabinose	35.5 (±0.6)	30.3 (±0.8)	1.17
L-gulose	53.3 (±1.1)	84.7 (±2.6)	0.63
D-lyxose	12.9 (±0.3)	66.8 (±2.6)	0.19

**Figure 5:**
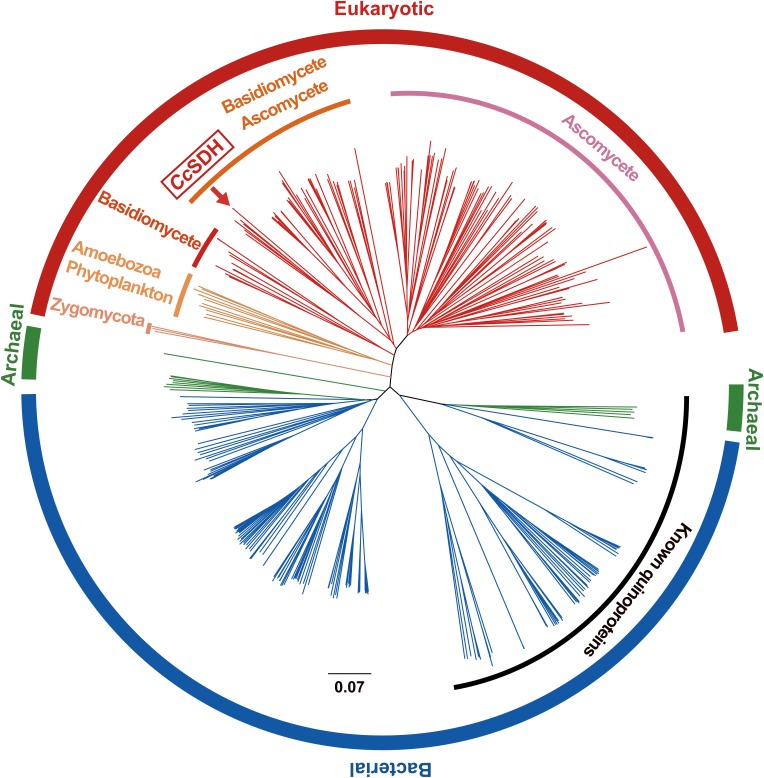
Phylogenetic tree of prokaryotic and eukaryotic quinoproteins. The phylogenetic tree was generated from the amino acid sequences of *Cc*SDH, proteins homologous to *Cc*SDH, and known quinoproteins using ClustalX (ver. 2.1) with the neighbor-joining method.

BLAST search revealed many genes that encode enzymes similar to *Cc*SDH in bacteria, archaea, amoebozoa, and fungi. Therefore, this type of quinoprotein appears to be widely distributed not only in prokaryotes, but also in eukaryotes. A phylogenetic tree generated from the amino acid sequences showed that these quinoproteins are distinct from other classes of known quinoproteins (Figure 5), suggesting that they represent a new family.

## Discussion

The highest amino acid homology to the unknown domain of *Cc*SDH was obtained with *Deinococcus radiodurans* L-sorbosone dehydrogenase (26%). Glucose dehydrogenase and sorbosone dehydrogenase, which have the highest degree of similarity to this protein, are both PQQ-dependent dehydrogenases. The catalytic activity for various sugars was observed only in the presence of PQQ. The strong binding activity of the protein to PQQ was shown by ITC. Based on these findings, we concluded that *Cc*SDH is a PQQ-dependent enzyme, even though it shows low amino acid sequence homology with known PQQ-dependent enzymes.

**Figure 6:**
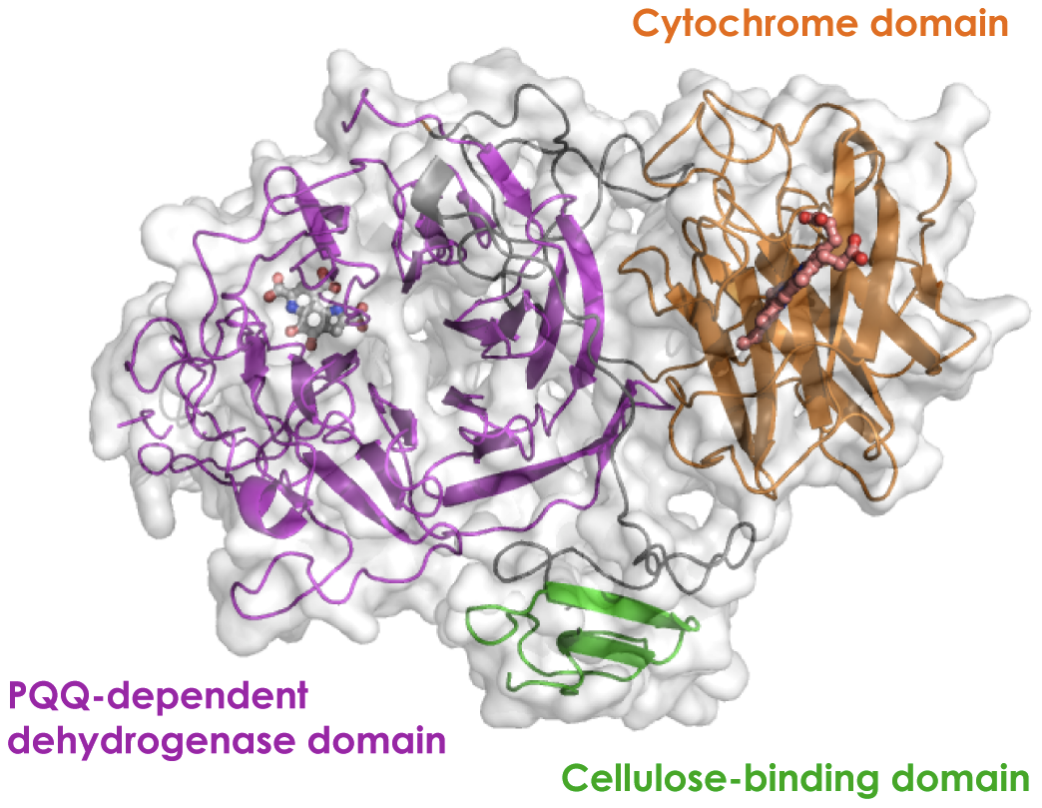
3D structure model of *Cc*SDH showing its three domains the N-terminal AA family 8 cytochrome domain (*orange*), the PQQ-dependent dehydrogenase domain (*magenta*), and the C-terminal cellulose-binding domain belonging to CBM family 1 (*green*). PQQ of sGDH from *Acinetobacter calcoaceticus* and heme in the cytochrome domain of *P. chrysosporium* CDH (PDBID 1CRU and 1D7C, respectively) were superimposed to the model.

**Figure 7:**
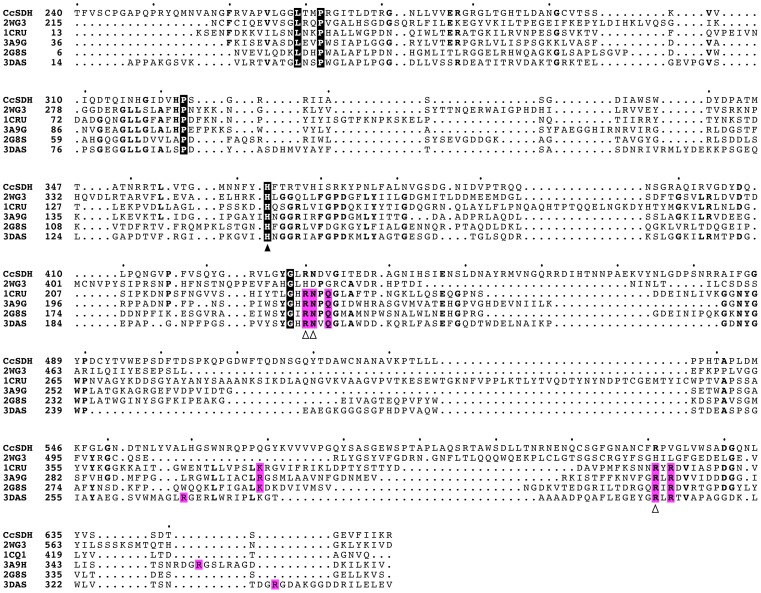
Alignment of the amino acid sequences of the catalytic domain of *Cc*SDH and known structure of six-bladed quinoproteins. Perfect matches are enclosed in boxes with a black background. Boxes with a magenta background indicate the amino acid residues interacting with PQQ via direct hydrogen bonds in known structures. The filled arrowhead indicates the position of the proposed catalytic histidine in bacterial quinoproteins. Conserved residues involved in PQQ-binding in known quinoproteins are indicated by open arrowheads if also conserved in *Cc*SDH [32]–[34].

The three-dimensional structures of PQQ-dependent quinoproteins are generally divided into two types: an eight-bladed beta-propeller structure with each blade consisting of a four-stranded anti-parallel beta-sheet, and a six-bladed beta-propeller structure [3], [29]. These two types of enzymes have no amino acid sequence homology with each other. The three-dimensional structure of *Cc*SDH was modeled using the Phyre2 protein fold recognition server and the predicted structure was compared with those of other quinoproteins and structurally similar proteins. *Cc*SDH was predicted to have a six-bladed instead of an eight-bladed quinoprotein structure (Figure 6). As shown in Table 2, *Cc*SDH shows some structural homology with known prokaryote PQQ-dependent sugar dehydrogenases, sGDH [30]–[32] and soluble aldose sugar dehydrogenases (Asd) from *Pyrobaculum aerophilum* [33] and *Escherichia coli* [34], although the amino acid sequence homology is low (≈20%). Among prokaryotic sugar dehydrogenases, *Cc*SDH shows some structural similarity with human hedgehog-interacting protein (HHIP) [35]. As seen in the alignment of the amino acid sequence of *Cc*SDH with those of known six-bladed quinoproteins (Figure 7), a putative catalytic histidine is conserved in all proteins, whereas several amino acids contributing to PQQ binding in the prokaryote sugar dehydrogenase are missing in *Cc*SDH and HHIP. However, as demonstrated above, the activity of *Cc*SDH is PQQ-dependent. Thus, the amino acids involved in the adsorption of PQQ should be different in this enzyme family, indicating the diversity of PQQ-binding motifs in eukaryotic enzymes.

**Table 2:** Summary of sequence homology modeling results using the Phyre2 server.

Enzyme	Organism	PDBID	Identity	Confidence	E-value¶
soluble glucose dehydrogenase (sGDH)	*Acinetobacter calcoaceticus*	1CRU	16	100.0	1.90E-34
aldose sugar dehydrogenase	*Pyrobaculum aerophilum*	3A9G	22	100.0	1.10E-28
soluble aldose sugar dehydrogenase (Asd)	*Escherichia coli*	2G8S	15	100.0	1.30E-26
human hedge hog interacting protein (HHIP)	*Homo sapiens*	2WG3	16	100.0	1.60E-27
putative glucose dehydrogenase	*Thermus thermophilus*	2ISM	16	100.0	9.00E-27
aldose sugar dehydrogenase (Adh)	*Streptomyces coelicolor*	3DAS	17	100.0	9.40E-24

¶ E-value generated using the Phyre2 profile-profile alignment algorithm.

The PQQ-dependent sugar dehydrogenase that we discovered here has interesting enzymatic properties associated with characteristic cytochrome (AA family 8) and cellulose-binding domains (CBM family 1) as well as the enzymes (proteins) related to plant cell wall degradation, and shows high activity towards rare sugars. Therefore, the discovery of the PQQ-dependent domain in *Cc*SDH could form the basis for a new AA family in the CAZy database. Detailed enzymatic studies will be necessary to evaluate why eukaryotes produce and secrete such enzymes, and how these enzymes acquire PQQ, because only a few prokaryotic organisms has been reported to be capable of synthesizing PQQ. Interestingly, genes encoding enzymes similar to *Cc*SDH were found in various bacteria, whereas only a few archaea contained corresponding genes. Furthermore, all bacterial and archaeal *Cc*SDH-like enzymes lack the cytochrome domain present in some of the fungal enzymes. This observation raises the question as to how eukaryotes such as fungi acquired genes encoding PQQ-dependent enzymes.

It is worth noting that this discovery would not have been possible using the usual approach of searching for PQQ-dependent enzymes. Our results indicate the existence of a previously unknown enzyme family. That PQQ is a beneficial vitamin for humans has been reported [36]. In view of our findings, novel PQQ-dependent enzyme(s) may exist in humans.
